# Failure Rates During Reuse of Disposable N95 Masks in Clinical Practice in the Emergency Department

**DOI:** 10.5811/westjem.2021.1.49960

**Published:** 2021-04-19

**Authors:** Ronald Check, Brian Kelly, Kathleen McMahon, Vamsi Balakrishnan, Leah Rivard, Johnathan Pester, Donald Jeanmonod, Rebecca K. Jeanmonod

**Affiliations:** St. Luke’s University Health Network, Department of Emergency Medicine, Bethlehem, Pennsylvania

## Abstract

**Introduction:**

The coronavirus 2019 pandemic caused a shortage of disposable N95 respirators, prompting healthcare entities to extend the use of these masks beyond their intended single-use manufacturer recommendation with a paucity of supporting research.

**Methods:**

We performed a prospective cohort study of ED healthcare workers (HCW) (“subjects”) required to use respirators at an academic, Level I trauma center. Subjects had been previously fit tested and assigned an appropriately sized N95 mask per hospital protocol. Per study protocol, subjects were fit tested periodically throughout their shifts and on multiple shifts over the eight-week study period. Data points collected included the age of the mask, subjective assessment of mask seal quality, and fit test results. We analyzed the data using Fisher’s exact test, and calculated odds ratios (OR) to determine the failure rate of disposable N95 masks following reuse.

**Results:**

A total of 130 HCWs underwent fit testing and 127 were included for analysis. Mask failure rate climbed after day 2 of use, with 33.3% of masks failing at day 3, 42.9% at day 4, and 50% at ≥ day 5. Categorizing the masks into those being used for two or fewer days vs those in use for three or more, failure was more common on day 3 of use or older compared to those in the first two days of use (41.8% vs 8.3%, P < 0.0001) with an OR of failure with an older mask of 7.9 (confidence interval [CI], 2.8–22.3). The healthcare workers’ assessment of poor seal was 33.3% sensitive (CI, 18.6–51.9) and 95.7% specific (CI, 88.8–98.6) for fit test failure.

**Conclusion:**

Disposable N95 masks have significant failure rates following reuse in clinical practice. Healthcare personnel also performed poorly in assessing the integrity of the seal of their disposable respirators.

## INTRODUCTION

In 1970 the United States Congress created the Occupational Safety and Health Administration (OSHA) to set forth regulations for workplace safety, as well as the National Institute for Occupational Safety and Health (NIOSH) to certify protective equipment and develop recommendations on its use.^1^ Initially used in industrial and manufacturing workplaces, disposable respirator masks were introduced in the healthcare setting to protect workers against airborne diseases. The tuberculosis outbreak of 1991 acted as a catalyst to prompt the US Centers for Disease Control and Prevention (CDC) to release guidelines for their use in healthcare facilities.^2^

Disposable respirator masks approved by NIOSH are designed as single- or limited-use respirators that an individual can mold to one’s face to ensure a proper seal and are intended for a single patient encounter. The NIOSH-certified N95 filtering facepiece respirator (FFR) is the most common disposable respirator used in the healthcare environment and is designed to fit securely on the operator’s face with the mask material meeting a minimum requirement of filtering 95% or greater of a standard test aerosol.^3^

The coronavirus 2019 pandemic quickly caused worldwide shortages in FFRs and other personal protective equipment (PPE).^4^ Supply chains and manufacturing have been hampered while demand has increased, and this imbalance in supply and demand will likely continue for some time. With critical shortages, it is neither economically nor logistically feasible to use disposable FFRs solely in their intended single-use capacity. Therefore, the CDC responded by publishing guidelines for healthcare entities to implement protocols for extended use of respirators (donning for multiple patient encounters without doffing), as well as limited reuse protocols (donning and doffing multiple times with the same mask for an extended period of time) to extend the lifetime of their supplies.^5^

Although this practice would stretch supplies for a longer period of time, there is a concern that extending the clinical use of disposable FFRs beyond their intended design could result in the reduction of protective effectiveness. The effectiveness of FFRs could be reduced by degradation of the filter medium itself or by loss of tight seal to the face such that not all inhaled air travels through the mask filter.^6^ Studies supporting mask reuse and extended use have been largely performed through simulated scenarios, with only one study to date addressing concerns of mask performance reliability with reuse and extended use.^7^–^9^

### Objectives

Our objectives were twofold: to determine the failure rate of disposable N95 FFRs reused over multiple days in the emergency department (ED), and to evaluate whether healthcare workers (HCW) were able to recognize the functional performance of their masks.

## MATERIALS AND METHODS

### Study Design

This was an anonymous, cross-sectional, convenience study of HCWs who were required to reuse disposable N95 FFRs during clinical duties in the ED. The study was reviewed by the institutional review board and found to be exempt.

### Study Setting and Population

The study was conducted from April 1–June 15, 2020 at a community-based Level I trauma center with an annual census of 55,000. Study subjects (hereafter referred to as HCWs) were physicians (both residents and attendings), nurses, medical technicians, and radiology technicians who had already been fit tested by the study site and assigned an appropriately sized N95 FFR as per OSHA mandate. During the study period, HCWs at the study site were required to wear N95 FFRs for the duration of their clinical shifts. We excluded HCWs who had failed institutional fit testing and were relegated to use a powered air-purifying respirator hood. We also excluded HCWs for whom no mask was available at the time of enrollment due to supply shortages, HCWs who declined participation, and those who provided their own PPE.

**Population Health Research Capsule**

What do we already know about this issue?

*The COVID-19 (coronavirus disease 2019)pandemic caused a worldwide shortage of disposable N95 respirators, prompting healthcare workers to reuse these masks beyond their intended single use.*

What was the research question?

*What is the failure rate of disposable N95 respirators following reuse in the emergency department?*

What was the major finding of the study?

*N95 respirators have significant failure rates following reuse, specifically after two days of use.*

How does this improve population health?

*Knowing that N95 respirators fail to provide adequate protection following reuse, healthcare systems can alter their masking policies to protect healthcare workers.*

### Study Protocol and Measurements

The HCWs wore a variety of N95 FFRs supplied by the hospital. These FFRs were either purchased by the hospital or donated to the hospital by outside organizations and then approved for clinical use after assessment by hospital resource management. Mask types included 3M 1860, 3M 8210, 3M Aura 1870 (3M Company, Saint Paul, MN), Kimberly-Clark 46727 (Kimberly-Clark Corporation, Irving, TX), Milwaukee 50-73-4010 (Milwaukee Electric Tool, Brookfield WI), and Honeywell H801 (Honeywell International, Inc, Charlotte, NC).

Prior to testing, HCWs recorded their impressions of the adequacy of their mask fit (adequate or inadequate) and total number of shifts during which their masks were worn. They subsequently underwent qualitative fit testing using a standardized hood and 3M FT-32 bitter testing solution (Bitrex). The HCWs performed standard maneuvers during fit testing, including breathing with their mouths open, rotating their heads side to side, tilting their heads up and down, and speaking. The fit test was performed by investigators who completed standardized OSHA training in fit-test performance. If the HCW tasted the bitter aerosolized solution during testing, he or she was considered to have failed the test and his or her mask was discarded and replaced with a new mask.

The results of fit testing were recorded on a standardized data collection sheet. Specific HCW role and further demographic data were not recorded. Some HCWs were tested once, while others were enrolled more than once with each new mask that they used. The number of times a HCW was enrolled was not recorded. Because of the large and changing variety of mask types approved by the hospital and used in clinical practice due to limited supplies, specific mask type was not recorded for subgroup analysis, as any particular mask design was unlikely to be used frequently enough to draw statically relevant conclusions.

### Data Analysis and Handling

The HCW’s impression of adequacy of mask fit and results of fit testing were recorded in a standardized spreadsheet by a single investigator. We analyzed data using descriptive statistics. Data for rate of mask failure as a factor of number of shifts worn was analyzed using Fisher’s exact test. We analyzed data regarding HCW accuracy in prediction of mask failure using chi square, with sensitivity and specificity analyses. All data was analyzed using MedCalc statistical software (MedCalc Software, Ostend, Belgium) and VassarStats.net, (a statistical computation website developed by Richard Lowry at Vassar College, Poughkeepsie, NY).

## RESULTS

**Figure 1:**
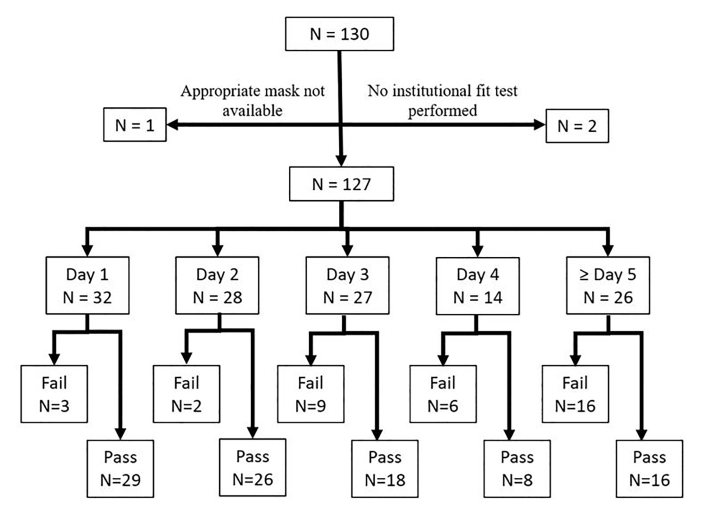
Flow diagram of study subjects demonstrating mask distribution based on day of use as well as “pass” and “fail” rates.

**Figure 2:**
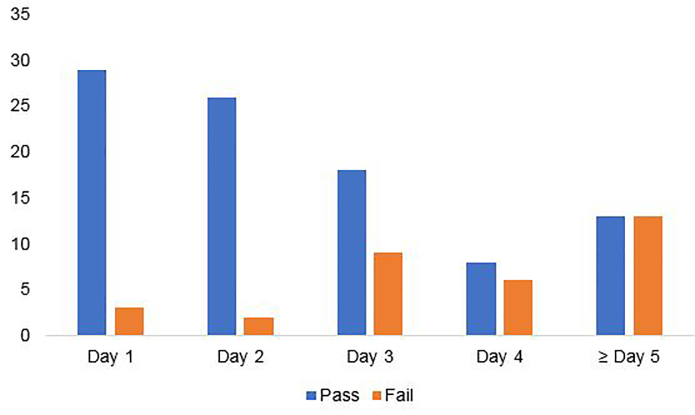
Graphical analysis of mask failure rates as a function of mask age, demonstrating a drop-off in mask pass rates beyond day 2.

A total of 130 HCWs underwent fit testing for the purposes of the study protocol. Two HCWs enrolled who had not been previously fit tested by the institution, and one HCW was wearing a mask that was not sized appropriately because of lack of supply of the previously tested mask. These three HCW were excluded from further analysis. Twenty-five percent of HCWs were on their first day of mask usage (n = 32), 22% were on their second day of mask usage (n=28), 21% were on their third day of mask usage (n = 27), 11% were on their fourth day of mask usage (n = 14), and 20% were on their fifth day or greater of mask usage (n = 26). The failure rate of masks was similar on the first and second day of usage at 9.4% and 7.1%, respectively (*P* = 1). Mask failure rate climbed after day 2 of use, with 33.3% of masks failing at day 3, 42.9% at day 4, and 50% at ≥ day 5 (Figure 1). Mask failure was more common in masks on day 3 of use or older compared to those in the first two days of use (41.8% vs 8.3%, *P* < 0.0001), with an odds ratio of failure of 7.9 (confidence interval [CI], 2.8–22.3)(Figure 2).

Fifteen HCWs felt that the seals on their masks were inadequate at the time of fit testing. Of these, 11 subsequently went on to fail their fit tests. Twenty-two HCWs who felt their masks had adequate seals failed their fit tests. HCW assessment of poor seal was 33.3% sensitive (95% CI, 18.6–51.9) and 95.7% specific (95% CI, 88.8–98.6) for fit test failure, with a positive predictive value of 73.3% (95% CI, 44.8–91.1), and a negative predictive value of 80.4% (95% CI, 71.6–87.0).

## DISCUSSION

In this study of mask failure rates in HCWs in clinical practice in an ED, mask failure rate climbed after day 2 of use with 41.8% of masks failing on day 3 of use or older. These results are consistent with the limited number of prior studies that examined the effect of multiple donning and doffing in extended use and reuse scenarios in simulated or laboratory scenarios. Bergman et al evaluated multiple N95 models and their fit over 20 consecutive donning and doffing episodes in a laboratory setting to simulate a single 10-hour shift of a HCW.^7^ Their findings suggested that HCWs were able to don masks five times with consistent passing, but beyond this number, there was a rise in failure rate.^7^ In spite of this, they noted that approximately 60% of FFRs had an adequate fit at the 20^th^ donning.^7^

Vuma et al evaluated fit factors of subjects undergoing six consecutive donning and doffing episodes.^8^ They found that 52% of subjects passed all six fit tests.^8^ However, half of those who failed returned to passing the fit test at some point.^8^ Sixteen percent of the subjects in their study failed persistently after the third fit test.^8^ In a more recent study, Degesys et al evaluated mask failure rates among HCWs in an ED over the course of three days.^9^ They found a failure rate of 38.2% with fit test failures associated with increased number of shifts that masks were worn, especially after day 2.^9^ We did not record total numbers of donning and doffing actions, but we believe it can be assumed that the number increases with the total number of shifts in which they are worn.

It is important to note that both Bergman et al^7^ and Vuma et al^8^ used quantitative fit testing during their studies, rather than qualitative testing, as was used in our study. In a study comparing Bitrex qualitative testing vs quantitative fit testing as the gold standard, the sensitivity and specificity of the Bitrex fit test was found to be 14% and 86%, respectively.^10^ This data indicates that the qualitative test is useful in identifying mask failures, but may result in identified failures at concentrations deemed acceptable by the quantitative method. These results may contribute to the slightly higher failure rates found both in our study and the study performed by Degesys et al, as compared to the studies using the quantitative method.^9^ Regardless of methods tested, studies demonstrate increased mask failure rates after prolonged use and re-use.

We did not assess whether our masks failed from loss of facial seal vs failure of the filter medium itself. A study performed by Grinshpun et al demonstrated that for N95 masks, the total particle penetration was between 2.5–5.5% depending on particle size, of which the majority was due to face seal leakage and <1% due to filter medium penetration.^11^ This suggests that our failures were likely due to seal failure and not medium failure. The same study also assessed the between-subject and within-subject variability in failure and found that 70% of total variability was associated with subject characteristics including facial size and shape and only 30% occurred due to donning.^11^ This finding indicates that although frequent donning and doffing may affect the mask seal quality, a person’s facial characteristics also contribute to the ability to adequately maintain an appropriate seal.^11^ We did not record data regarding face size and shape for our HCWs, but this may well have played a role in our mask failures.

The OSHA guidelines recommend a user-performed seal test with every donning of a respirator, which implies that this may be a reasonable screen for mask failures. Our HCWs are all trained in user-performed seal tests. However, our study suggests that HCWs have inadequate recognition of when they have a mask failure. In our population, if we relied only on self-assessment of seal, some masks would have been discarded that were still working appropriately, thereby wasting masks. Conversely, we would have missed a number of failures, potentially placing HCWs at risk.

Although exact infection rate in our department is not known, we are aware of only two documented cases of infection in ED HCWs over the testing period. This is substantially lower than expected given our high mask failure rates. We speculate that this may be secondary to our universal masking policy of all patients. In addition, the minimal infectious dose of COVID-19 remains unknown. It may be possible that even ill-fitting N95 masks offer enough protection to prevent infection. Secondly, the current estimates of asymptomatic infections are in the range of 40–45% with individual studies documenting asymptomatic cases between 6.3–96%.^12^ As our facility does not currently have universal testing policies in place for HCWs, it is plausible that we have had more infections than reported. Additionally, it is possible that “failed” masks intermittently had adequate fit, as one study has shown that 50% of failed masks returned to fit later on in the study on re-testing.^7^ Therefore, some failed masks might have provided adequate seal prior to testing, and the seal was only broken during provocative testing occurring during testing, but not during clinical duties.

## LIMITATIONS

Our study is limited by its anonymous design, which precludes our ability to determine whether failures were more common among different types of HCWs (for example, nurses as compared to technicians). Additionally, because of the variety of masks approved by and used through hospital resource management, we did not record whether a specific disposable mask was more likely to fail than another. Anecdotally, there were failures in all groups, but the numbers of each mask were too low to draw conclusions for significance between the groups. We relied upon self-report regarding age of mask. In our institution, HCWs place a hash mark on the outside of the mask with a permanent marker for each day the mask is in use, but it is possible the HCW might have forgotten to mark the mask on a given day, thereby underestimating the age of the mask.

We did not control for number of donning and doffing episodes, which makes our results more difficult to compare to other studies. However, we felt reports of the number of donning and doffing episodes in clinical practice (as opposed to in simulated scenarios) would likely be unreliable, and that HCWs might inconsistently define donning and doffing episodes. (For instance, we witnessed HCWs briefly pull down a mask to drink or speak who stated they had worn their masks continuously.) We also did not control for method of decontamination of mask between shifts. Although our institution provides UV decontamination for disposable masks, it is possible that some HCWs chose not to avail themselves of this service, and might have used other methods, such as simply not using the mask for a few days. Likewise, we did not query HCWs as to the storage and care of their masks between uses. Finally, our study was performed at a single institution, and may not be generalizable to all settings.

## CONCLUSION

Reuse of disposable filtering facepiece respirators beyond two days in actual clinical practice has a high rate of fit failure. This suggests increased risk of aerosolized infectious disease transmission with reuse of masks. This risk might be mitigated with frequent fit testing. Healthcare workers perform poorly in recognizing the integrity of their own mask seals. Therefore, self-assessment does not appear adequate to determine fit.
